# Characteristics and outcomes of pediatric patients presenting at Cambodian referral hospitals without appointments: an observational study

**DOI:** 10.1186/s12245-018-0172-0

**Published:** 2018-03-13

**Authors:** Mackensie A. Yore, Matthew C. Strehlow, Lily D. Yan, Elizabeth A. Pirrotta, Joan L. Woods, Koy Somontha, Yim Sovannra, Lauren Auerbach, Rebecca Backer, Christophe Grundmann, Swaminatha V. Mahadevan

**Affiliations:** 1 Department of Emergency Medicine, UCSF Fresno Center for Medical Education and Research, 155 N Fresno St, Fresno, CA 93701 USA; 2 Department of Emergency Medicine, Stanford University School of Medicine, 300 Pasteur Drive, Stanford, CA 94305 USA; 3 Department of Internal Medicine, Boston University Medical Center, Boston, MA USA; 4 USAID Central Asia, Almaty, Kazakhstan; 5 University Research Co., LLC, Centre for Human Services, Phnom Penh, Cambodia; 6 GIZ-Social Health Protection Program Cambodia, Phnom Penh, Cambodia; 7 University of Arizona College of Medicine—Phoenix, Phoenix, AZ USA; 8 UCSF Benioff Children’s Hospital Oakland, Oakland, CA USA

**Keywords:** Chief complaints, Developing countries, Emergency medicine, Health systems, Pediatrics

## Abstract

**Background:**

Emergency medicine is a young specialty in many low- and middle-income countries (LMICs). Although many patients seeking emergency or acute care are children, little information is available about the needs and current treatment of this group in LMICs. In this observational study, we sought to describe characteristics, chief complaints, management, and outcomes of children presenting for unscheduled visits to two Cambodian public hospitals.

**Methods:**

Children enrolled in the study presented without appointment for treatment at one of two Cambodian public referral hospitals during a 4-week period in 2012. Researchers used standardized questionnaires and hospital records to collect demographic and clinical data. Patients were followed up at 48 h and 14 days after initial presentation. Multivariate logistic regression identified factors associated with hospital admission.

**Results:**

This study included 867 unscheduled visits. Mean patient age was 5.7 years (standard deviation 4.8 years). Of the 35 different presenting complaints, fever (63%), respiratory problems (25%), and skin complaints (24%) were most common. The majority of patients were admitted (51%), while 1% were transferred to another facility. Seven patients (1%) died within 14 days. Follow-up rates were 83% at 48 h and 75% at 14 days. Predictors of admission included transfer or referral from another health provider, seeking prior care for the presenting problem, low socioeconomic status, onset of symptoms within 24 h of seeking care, abnormal vital signs or temperature, and chief complaint of abdominal pain or fever.

**Conclusions:**

While the admission rate in this study was high, mortality was low. More effective identification and management of children who can be treated and released may free up scarce inpatient resources for children who warrant admission.

**Electronic supplementary material:**

The online version of this article (10.1186/s12245-018-0172-0) contains supplementary material, which is available to authorized users.

## Background

In low- and middle-income countries (LMICs), an estimated 45% of all deaths and 36% of disability-adjusted life years (DALYs) are due to diseases and injuries typically addressed by emergency medical services that are currently lacking in these regions [1]. Among these countries is Cambodia, a lower middle-income nation in Southeast Asia which still suffers residual effects of the Khmer Rouge regime of the 1970s, during which violence, famine, and preventable disease killed approximately two million people and completely dismantled the healthcare infrastructure [2, 3].

Developing emergency medicine in LMICs requires a great deal of specialized training for providers. Characterizing the potential emergency department population in LMICs is an important step in creating relevant training for future emergency clinicians. Understanding the distribution of chief complaints focuses provider education on symptoms with the highest burden of morbidity and mortality, thereby helping emergency care providers more effectively evaluate and manage patients [4]. Despite the importance of this information, the 2013 Academic Emergency Medicine Consensus Conference found insufficient data on chief complaints for most LMICs [4]. Helping address this knowledge gap, our group previously documented the epidemiology and outcomes of adult patients presenting for unscheduled visits to public hospitals in Cambodia [5]; no studies, however, have thus examined an analogous pediatric population in this setting.

Pediatric patients account for 20–35% of all emergency department (ED) visits globally [6–8]. Moreover, children present with a unique distribution of chief complaints compared to adults and require different clinical management [7, 9]. To address the specific needs of children presenting to EDs in LMICs, the World Health Organization (WHO) developed guidelines for the care of children with traumatic injuries and acute illnesses in resource-limited settings; however, limited training for diagnosing and treating urgent medical conditions poses challenges to guideline adherence, which may contribute to preventable morbidity and mortality in LMICs [10–15]. The recently announced United Nations Sustainable Development Goals includes a call to reduce preventable mortality among children; strengthening pediatric emergency care capacity in LMICs could help achieve this goal. The present study was conducted to identify the characteristics, chief complaints, management, and outcomes of children presenting for unscheduled visits to two public referral hospitals in Cambodia in order to focus training to improve future care.

## Methods

### Study design and setting

We performed a 4-week prospective, cross-sectional study of unscheduled visits to two government provincial referral hospitals in Cambodia: Sampov Meas Provincial Hospital (SMPH) and Battambang Provincial Hospital (BPH). SMPH and BPH are “CPA Level 3,” indicating that obstetric, emergency, and surgical services should be available [16]. SMPH has 162 inpatient beds and recorded 9722 visits in 2012, leading to 6564 admissions. BPH has 220 inpatient beds and recorded 40,825 visits and 14,108 admissions in 2012 [17].

The level of emergency care offered at these two hospitals was similar to that at other Cambodian provincial hospitals at the time of the study. Neither hospital had an active triage system, and BPH had no ED, with patients presenting to a variety of departments for treatment. At BPH, most pediatric patients seeking urgent care came directly to the pediatric ward. At SMPH, the hospital’s combined ED/ICU managed patients in serious condition, while patients with milder presentations presented elsewhere. At both facilities, study enrollment was hospital wide.

### Selection of participants

Pediatric patients (< 18 years) presenting without prior appointments during the 4 weeks in July and August 2012 were invited to enroll; patients presenting for routine check-ups and vaccinations without appointment were excluded. Repeat unscheduled visits were considered separate visits. Most enrollment occurred weekdays, 08:00 to 17:00, times of day with highest patient volume. Patients presented infrequently during evening and weekend hours when hospital staffing was limited. Afterhours visits were included if the patient remained at the hospital the following morning.

### Methods and measurements

A team at each site gathered real-time clinical and demographic data using Research Electronic Data Capture (REDCap) forms [18]. Demographic information and up to three chief complaints were obtained from patients, guardians, and staff. A list of all chief complaints is included in the supplemental material (Additional file 1: Figure S1). Hospital records provided vital signs at presentation, diagnostic tests, treatment interventions, disposition, and discharge dates. Two members of the research team reviewed records for completeness and inconsistencies, which were addressed through repeat interviews and hospital record reviews. Follow-up interviews with patients or their guardians were conducted in Khmer (the local language) at 48 h and 14 days following the initial visit. Follow-up interviews were in-person if the patient remained hospitalized, or by telephone if discharged, and assessed patient location, survival, and functional status. Patients were considered lost to follow-up after three failed contact attempts on successive days.

### Outcome measures

Primary outcomes included admission, functional impairment, and mortality. Patients are typically treated for their entire hospital stay in the department where they initially presented, regardless of appropriateness; therefore, included interventions were limited to those completed within the first 48 h of initial presentation, thus identifying services most representative of a standard ED setting. Patients staying overnight were considered admitted. Functional impairment was defined as continued hospitalization, significant pain or limitation in performing daily activities, bed confinement, or coma.

### Data analysis

Because a complete set of vital signs was not consistently documented at participating hospitals, records with data missing for respiratory rate (RR), blood pressure (BP), or heart rate (HR) were included in multivariate analyses with absent values assumed normal. Records with missing values for all other independent variables were excluded from modeling. Sensitivity analysis showed no significant differences in predictors of admission with the conversion of missing vital signs to normal in multivariate analysis.

Comparisons between outcomes for continuous variables were conducted using Wilcoxon two sample *T* test, while the chi-square test was used for categorical variables. A multivariate logistic regression was built for the primary outcome of admission using predictors identified through univariate analysis, controlling for age and gender; stepwise methods were not used. Statistical analysis was completed using SAS Enterprise Guide for Windows, version 4.3 (SAS Institute Inc. Cary, NC).

### Ethical considerations

We obtained verbal informed consent from patient guardians or patients themselves if unaccompanied and at least 16 years old. In-person, native-speaking translators obtained consent and conducted interviews in Khmer.

The Institutional Review Boards at Stanford University School of Medicine (IRB-24735) and the Cambodian Ministry of Health approved this study.

### Availability of data and materials

The dataset supporting the conclusions of this article is available in the Dryad repository, 10.5061/dryad.7v8c4.

## Results

### Demographic characteristics

This 4-week study documented 867 unscheduled pediatric patient visits. Mean patient age was 5.7 years with approximately half (54.3%) being male (Table 1). Private vehicles were the predominant mode of transport to the hospitals, with nearly 90% of patients arriving via motorbike, taxi, or tuk-tuk (motorized three-wheeled rickshaw for hire); arrival by ambulance was infrequent (2.9%). More than 69.9% of patients presented to the hospital with sudden (< 24 h) or recent (1–3 days) symptoms (Table 1). About one quarter of patients were either transferred directly from another healthcare facility, typically a health center, or referred by an outside medical provider (e.g., a practitioner private clinic) (Table 1).

**Table 1:** Pediatric patients presenting unscheduled at two Cambodian hospitals, July–August 2012

Characteristic	No. (%) of patients^a, b^
All patients	867
Demographic characteristic	
Site of presentation	
Battambang Provincial Hospital	550 (63.4)
Sampov Meas Provincial Hospital	317 (36.6)
Age	
Age in years, mean (SD)	5.65 (4.8)
Infant (< 1 year)	136 (15.7)
Young child (1–5 years)	374 (43.1)
Child (6–10 years)	182 (21.0)
Older child (11–13 years)	98 (11.3)
Teen (14–17 years)	77 (8.9)
Gender	
Female	396 (45.7)
Male	471 (54.3)
Socioeconomic characteristic	
Patient had low-income health insurance^c^	444 (51.2)
Travel to hospital	
Time	
Time in hours, median (IQR)	0.5 (0.3–1.0)
Time < 0.5 h	482 (55.6)
Time 0.5–2 h	326 (37.6)
Time > 2 h	52 (6.0)
Distance	
Distance in kilometers, median (IQR)	10 (3.0–30.0)
Presentation	
Time of arrival	
Daytime Monday to Friday (i.e., 07.00–17.00)	765 (88.2)
Overnight Sunday to Friday (i.e., 17.00–07.00)	68 (7.8)
Weekend (i.e., Friday 17.00 to Sunday 17.00)	34 (3.9)
Care before presentation	
Transferred from another healthcare facility	163 (18.8)
Referred by an external medical provider	81 (9.3)
Prior care of those not transferred or referred	
Prior care received in the previous 48 h	79 (9.1)
Prior care received more than 48 h earlier	65 (7.5)
No prior care	468 (54.0)
Symptom duration	
Sudden (< 24 h)	266 (30.7)
Recent (1–3 days)	340 (39.2)
Sub-acute (4–14 days)	206 (23.8)
Chronic (> 14 days)	49 (5.7)
Time before seeking any medical care	
Median time before seeking care in days (IQR)	1.5 (0.6–3)
< = 24 h	339 (39.1)
> 24 h	505 (58.3)
Examination findings	
Respiratory rate, blood pressure, and heart rate^d^	
Abnormal respiratory rate, blood pressure, or heart rate	405 (46.7)
Abnormal respiratory rate	224 (25.8)
Abnormal blood pressure	153 (17.6)
Abnormal heart rate	217 (25.0)
Respiratory rate, blood pressure, or heart rate not recorded	216 (24.9)
Temperature	
Low temperature (< 36 °C)	80 (9.2)
High temperature (> 38 °C)	153 (17.7)
Temperature not recorded	213 (24.6)
Pain	
Pain documented as present	254 (29.3)

*IQR* interquartile range, *SD* standard deviation

^a^Number of patients and percentages, unless otherwise stated

^b^Due to missing values for individual patients, not all categories sum to 100%

^c^Except for more affluent patients who had private health insurance, most patients paid in cash

^d^See Additional file 2: Table S2 for age-appropriate vital signs used

### Patient presentations and management

**Fig. 1:**
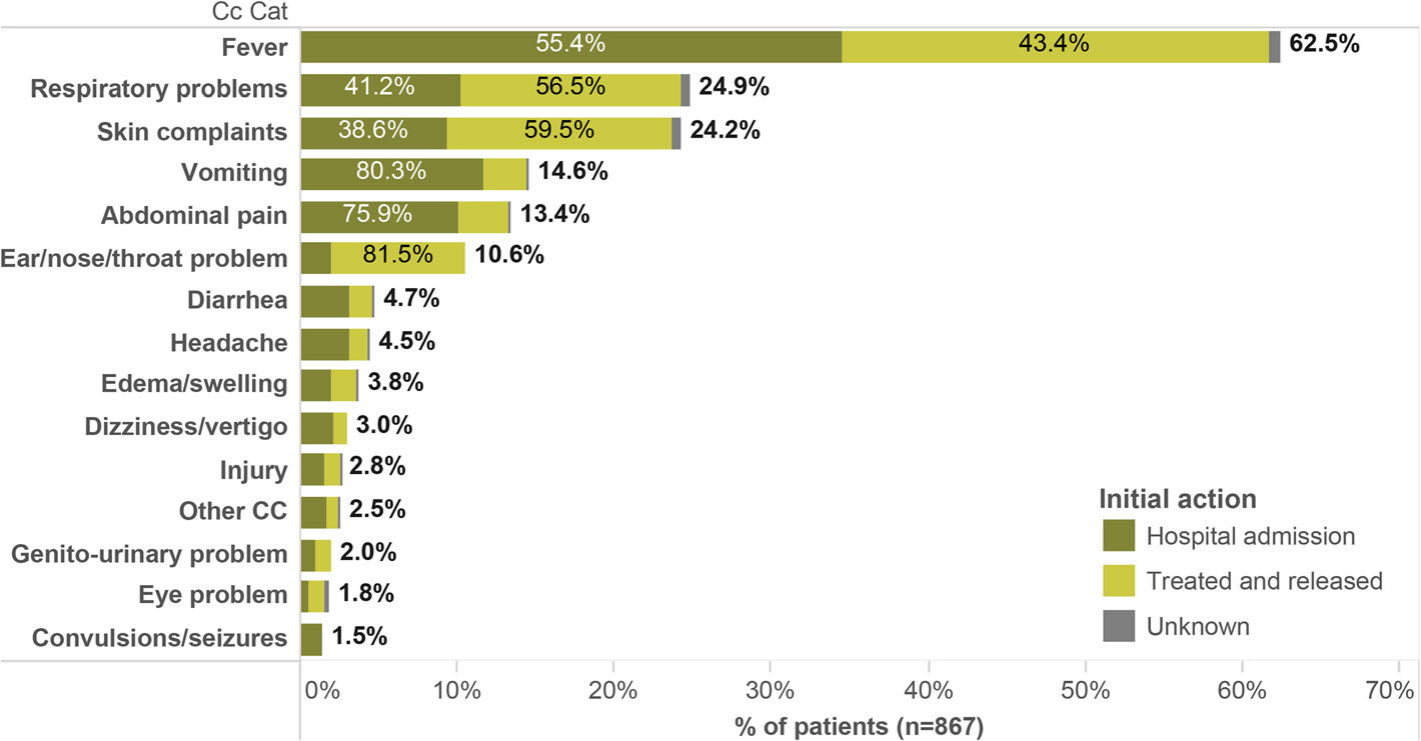
Chief complaints and initial actions for children presenting without appointment at two Cambodian hospitals. Only the top 15 chief complaints are displayed. A maximum of three chief complaints per patient were recorded. Includes data for July–August 2012

For the 867 pediatric patients enrolled, 1615 total chief complaints were recorded, 35 of them distinct. Fever was the most common chief complaint (62.5% of patients; 33.6% of complaints), followed by respiratory problems, skin-related complaints, vomiting, and abdominal pain (Fig. 1). Injury represented 2.8% of complaints (Fig. 1). Chief complaints of abdominal pain and fever resulted in a significantly higher admission rate (*p* ≤ 0.05), while respiratory problems and skin complaints correlated with lower admission rates (*p* ≤ 0.01). Of the seven deaths, chief complaints included respiratory problems (3 children), vomiting (3), fever (2), unconsciousness (1), convulsions/seizures (1), genital bleeding (1), and other (1).

Approximately one quarter of all patient records lacked vital sign data (Table 1). Patients treated and released were less likely to have vital signs recorded than those admitted (*p* ≤ 0.05). Abnormal vital signs were more frequent in admitted patients than those treated and released (*p* ≤ 0.05); similarly, admitted patients were more likely to present with pain (*p* ≤ 0.05).

One third of patients received any diagnostic testing, with laboratory tests being the most common (Table 2). Imaging, including chest X-ray, and other diagnostics were performed on less than 3% of patients (Table 2). The most common intervention within the first 48 h was medication therapy, administered to 81.6% of patients (Table 2). Intravenous fluids were provided to 40.7% of patients. All other interventions were infrequent, each received by less than 5% of patients (Table 2).

**Table 2:** Diagnostics and interventions within 48 h in children presenting unscheduled at two Cambodian hospitals

Diagnostic test or intervention	No. (%) presenting^a, b^(*n* = 867)	No (%) admitted^b, c^(*n* = 445)
Diagnostic test		
Any test	278 (32.1)	263 (59.1)
Laboratory test	270 (31.1)	259 (58.2)
Diagnostic imaging	19 (2.2)	14 (3.2)
Diagnostic peritoneal lavage	2 (0.2)	2 (0.5)
Ultrasound scanning	1 (0.1)	1 (0.2)
Medication administered		
Any medication	707 (81.6)	343 (77.1)
Analgesic (excluding aspirin)	602 (69.4)	288 (64.7)
Antibiotic	412 (47.5)	172 (38.7)
Antiparasitic	62 (7.2)	7 (1.6)
Drug administered by nebulizer	29 (3.3)	18 (4.0)
Antimalarial	7 (0.8)	7 (1.6)
Aspirin	3 (0.4)	0
Antituberculosis drug	1 (0.1)	0
Other	255 (29.4)	94 (21.1)
Other interventions		
Any intervention other than medication	380 (43.8)	356 (80.0)
Intravenous fluids	353 (40.7)	345 (77.5)
Intravenous medication	42 (4.8)	41 (9.2)
Emergency cooling	35 (4.0)	35 (7.9)
Oxygen therapy	33 (3.8)	29 (6.5)
Oral hydration	29 (3.3)	19 (4.3)
Wound closure	13 (1.5)	11 (2.5)
Urethral catheterization	6 (0.7)	6 (1.3)
Gastric decontamination	6 (0.7)	6 (1.3)
Dental procedures	4 (0.5)	0
Blood transfusion	3 (0.3)	2 (0.4)
Endotrachial intubation	3 (0.3)	3 (0.7)

^a^Categories may sum to more than 100% because some patients had more than one diagnostic test or intervention

^b^Overall, 26 (3.0%) patients did not undergo any diagnostic test or receive any intervention and, for 82 (9.5%) of patients, no information on diagnostic tests or interventions was collected

^c^Overall, four (0.9%) admitted patients did not undergo any diagnostic test or receive any intervention and, for 49 (11.0%) patients, no information on diagnostic tests or interventions was collected

### Patient disposition and outcomes

The rate of admission was 51.3%, with a 3-day median length of stay (Table 3). Less than 1% of patients were transferred to an outside facility or left the hospital prior to being seen or against medical advice (Table 3). Twenty-two patients (3.1%) were referred to surgery, 81.8% of whom were admitted.

**Table 3:** Outcomes and follow-up in children presenting unscheduled at two Cambodian hospitals, July–August 2012

Outcome	No. (%) presenting (*n* = 867)^a^
Initial visit	
Patient treated and released	403 (46.5)
Patient transferred to another facility^b^	8 (0.9)
Patient left hospital without being seen or against medical advice	4 (0.5)
Patient died within 24 h of presentation	2 (0.2)
Patient admitted	445 (51.3)
Length of stay of patients admitted in days, median (IQR)	3 (2–4)
48-h follow-up	
Patient followed up at 48 h	719 (82.9)
Patient remained functionally impaired^c, d^	362 (50.3)
Patient seen by another healthcare provider after discharge^c^	45 (5.6)
Patient died between 24 and 48 h^c^	4 (0.6)
14-day follow-up	
Patient followed up at 14 days	649 (74.9)
Patient remained functionally impaired^d, e^	44 (6.8)
Patient seen by another healthcare provider after discharge^e^	86 (13.3)
Patient died between 48 h and 14 days^e^	1 (0.2)
Cumulative outcomes	
Patient had any surgical procedure^e^	22 (3.4)
Cumulative mortality at 14 days^e^	7 (1.1)

*IQR* interquartile range

^a^Number presenting and percentage, unless otherwise stated

^b^Includes only patients transferred to another facility without first being admitted and receiving care

^c^Percentage of the 719 patients followed up for 48 hours

^d^Functional impairment was defined as significant pain, significant limitation in performing daily activities, confinement to bed, or a comatose state

^e^Percentage of the 649 patients followed up for 14 days

Follow-up rates were 82.9 and 74.9% at 48 h and 14 days, respectively (Table 3). Seven deaths were documented, for a 1.1% overall 14-day mortality (Table 3). Two of the seven deaths occurred within 24 h of presentation; the rest occurred after admission, one of whom died after discharge. No deaths were documented in patients who were treated and released.

Morbidity was measured by asking patients or guardians whether the patient had returned to baseline functioning. By 40 h and 14 days post-visit, 49.7 and 93.2% of patients, respectively, had returned to baseline functioning (Table 3).

### Admission multivariate logistic regression model

A multivariate logistic regression model showed increased admission risk was associated with referral from another health provider (OR = 4.3, CI 2.4–7.8), direct transfer from another health provider (OR = 7.8, CI 4.7–13.0), low-income health insurance (proxy for low socioeconomic status) (OR = 1.5, CI 1.1–2.1), seeking care prior to 48 h before presentation without transfer/referral (OR = 2.4, 95% CI 1.–4.5), seeking care within 48 h of presentation without transfer/referral (OR = 2.5, CI 1.4–4.5), symptom onset within 24 h of seeking care (2.1, CI 1.5–3.1), abnormal temperature (OR = 1.5, CI 1.0–2.2), abnormal heart rate, respiratory rate, and/or blood pressure (OR = 3.2, CI 2.3–4.6), and chief complaint of abdominal pain (OR = 2.9; 95% CI 1.7–5.0) or fever (OR = 1.4, CI 1.0–2.0); a decreased risk of admission was associated with skin-related chief complaints (OR = 0.4; CI 0.2–0.5), such as rashes or blisters, and respiratory problems (OR = 0.4; CI 0.3–0.6), such as cough (*n* = 793; *c*-statistic = 0.81).

### Dermatologic and fever complaints sub-analysis

**Fig. 2:**
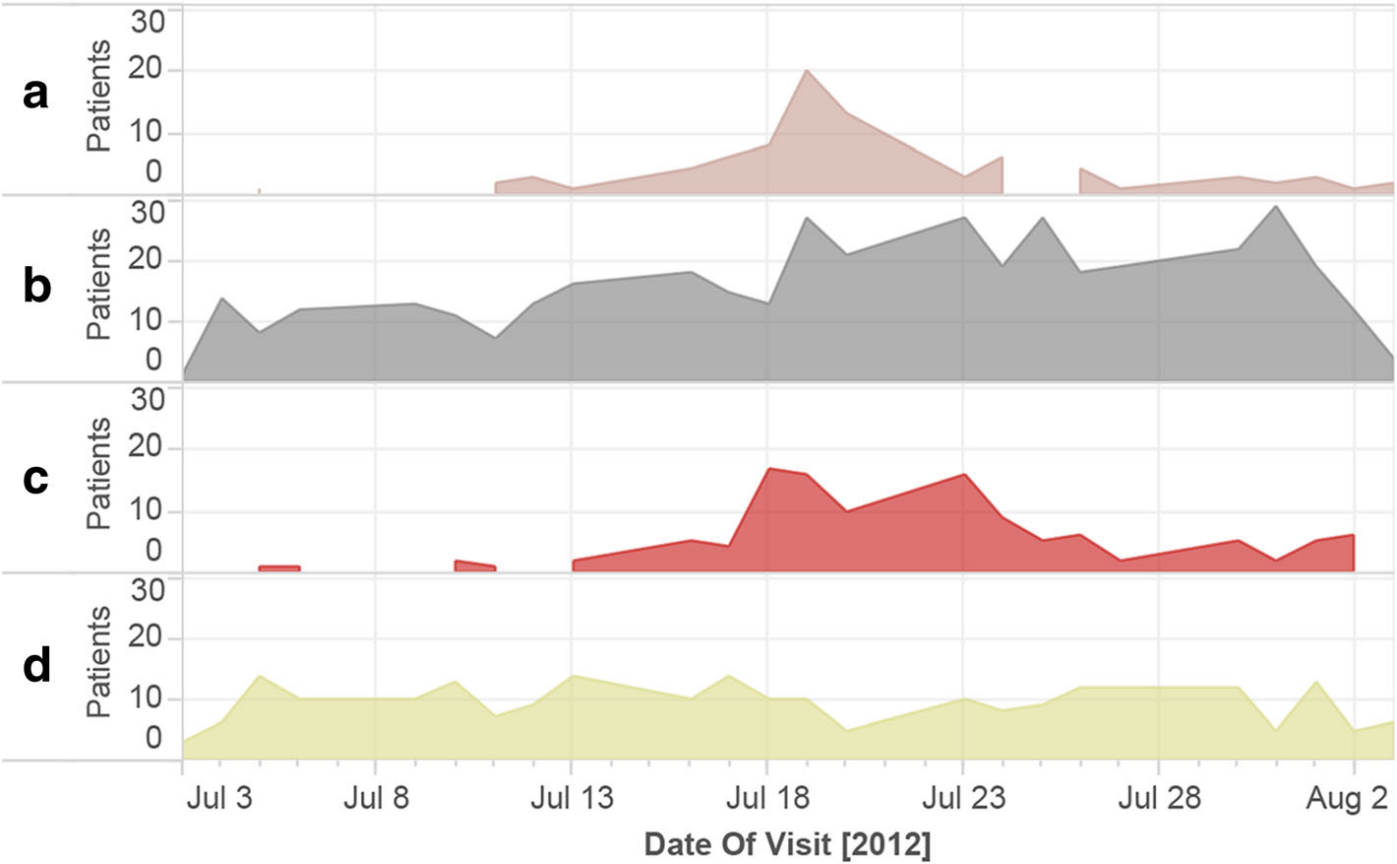
Presentation of children with specific chief complaints. **a** Skin-related chief complaints without fever complaint. **b** Chief complaint of fever without skin-related complaint. **c** Both skin-related and fever chief complaint. **d** All other chief complaints other than skin-related or fever. Patients who presented during weekends are not included, due to low weekend enrollment

A country-wide outbreak of enterovirus strain EV-71 occurred during the study. Qualitatively, many more children than expected presented with fever and/or skin complaints, such as rash or blisters, which are common symptoms of EV-71. The number of patients presenting with skin complaints and fever increased sharply during the study period before gradually decreasing, coinciding with timing of the EV-71 outbreak (Fig. 2).

## Discussion

This study describes the characteristics, chief complaints, management, and health outcomes of children seeking unscheduled acute care at two public referral hospitals in Cambodia. Our findings provide essential information to inform emergency care provider education and guide development of emergency care systems in Cambodia and other LMICs.

Top chief complaints in this study (fever, respiratory problems, abdominal pain, vomiting, and diarrhea) are similar to other published reports characterizing unscheduled or ED pediatric presentations in other LMICs [6, 8, 19, 20]. The top five chief complaints in our population accounted for 75% of all reported complaints. In contrast to several other studies, the percentage of ED visits attributed to trauma in this study (2.8%) was substantially lower. For example, trauma among children presenting for emergency care accounted for 27–29% of visits in Korea and Malaysia and 25% of visits in the USA [8, 21, 22]. The underrepresentation of trauma in our study may have been due to the presence of an NGO-run trauma hospital near the study sites, which might have received the bulk of trauma patients. The top chief complaints in our and other studies often have infectious etiologies [23, 24]. While infections such as pneumonia, diarrhea, and malaria are the leading contributors to mortality in children under 5 years worldwide, [25] infectious symptoms also account for a large percentage of ambulatory care-sensitive conditions [26].

Of the top ten chief complaints identified in the present study, only half match the list of top complaints documented in an analogous population of adults [5]. These results mirror data from EDs in the USA, which similarly show that the frequency and distribution of pediatric chief complaints differ markedly from adults [9]. The unique distribution and frequency of presentations in children with undifferentiated conditions compared to adults in LMICs underscores the need for specialty training that specifically addresses pediatric emergencies.

Regarding pre-hospital care, only 2.9% of patients in our study arrived by ambulance, compared to 15–17% in other studies in LMICs [27, 28]. Low frequency of ambulance transport—despite more than one quarter of all unscheduled patients coming from another care provider—reflects the relative underdevelopment of Cambodia’s regional pre-hospital and EMS systems. Currently, laypeople provide most patient transfers in Cambodia. Low-cost, community-based interventions aimed at equipping laypeople with first aid skills and facilitating transport have shown promise in South Africa [29].

Vital signs, which provide low-cost, objective data that can help prioritize patients during triage and guide initial therapy, were not measured or recorded in nearly one quarter of enrolled patients. Emphasis on obtaining vital signs should be included in all training for emergency care providers to improve management for pediatric patients presenting for acute care.

We also observed that respiratory complaints and fever were the two most common complaints, yet diagnostic chest X-rays (CXR) were infrequently performed, even for admitted patients. Furthermore, despite the high prevalence of tuberculosis in Cambodia (817/100,000 population) relative to other countries in the South-East Asia Region [30], only one patient received treatment for tuberculosis. These findings indicate that further studies are needed to assess the diagnostic evaluation among children with respiratory complaints to ensure that tuberculosis and other respiratory illnesses are recognized and adequately treated.

Finally, we observed a high admission rate (51.3%) compared to other published pediatric studies (15–35%) [8, 19, 20]. Since our 48-h mortality rate, a marker of patient severity, of 0.7%, was comparable to the 24-h mortality rate reported in other studies examining similar patient populations, it is likely that a portion of the admitted patients did not require inpatient care [20, 27]. Inappropriate admissions unnecessarily consume healthcare resources and place patients at increased risk for hospital-acquired infections, a known hazard in LMICs [31]. Such a high admission rate coupled with a relatively low mortality rate reinforces the need for a more organized emergency and acute care system in which providers are equipped to rapidly initiate diagnostic workup, provide timely treatment, and make informed decisions about patient disposition.

Our study found a high frequency of skin complaints and fever among enrolled patients relative to other studies. This was likely associated with the concurrent, widely publicized outbreak of a severe strain of enterovirus, EV-71. Symptoms included fever, rash or blisters inside the mouth and on the hands and feet, and, in severe cases, encephalitis and respiratory distress [32]. In mid-July 2012, the Cambodian Ministry of Health organized public education campaigns on symptoms of EV-71, recommending medical care for children with severe symptoms [33, 34]. The increase in patient visits during the outbreak, particularly during the peak stretch from 14 July 2012 to 25 July 2012, suggests that these campaigns effectively increased health-seeking behavior among patients with relevant symptoms.

### Limitations

Key limitations of this study included inability to capture seasonal variations in illness patterns during the 4-week study period or regional variation due to the close proximity of study hospitals to one another, the likely impact of the concurrent outbreak of EV-71, and lack of data collected on weekends and overnight. As stated above, the low frequency of trauma complaints at our study sites may be due to a nearby NGO-run hospital, and, therefore, is not generalizable across Cambodia.

## Conclusions

As far as we know, this is the first paper to report on the epidemiology of pediatric patients presenting unscheduled to hospitals in Cambodia. This paper builds on previous work characterizing the unscheduled adult population and finds important differences. Since pediatric patients have a different spectrum and frequency of chief complaints from adults in emergency care settings, this paper finds a need for both dedicated research into the unique presentations of children and specific training for medical professionals for emergency pediatric care. For next steps, results presented here can help inform the development of chief complaint-oriented training modules for medical providers who will staff newly founded emergency departments in Cambodia and similar settings. Results may also be useful to Cambodian hospital administrators and public health officials to further inform resource allocation for patient care, for example, by ensuring hospitals have sufficient equipment for measuring vital signs or investing in the implementation of a triage system. In addition, some specific results from this study may also serve as baseline metrics for patient outcomes—including admission rate, morbidity, and mortality—against which progress can be measured as emergency medicine matures as a distinct specialty and practice in Cambodia and other LMICs.

## Additional files

Additional file 1: **Figure S1.** Chief complaints. All chief complaints reported by patients were coded by researchers as one of the complaints listed. (PDF 1287 kb)

Additional file 2: **Table S1.** Criteria for abnormal vital signs. (PDF 171 kb)

## Abbreviations

BP: Blood pressure
BPH: Battambang Provincial Hospital
DALYs: Disability-adjusted life years
ED: Emergency department
HR: Heart rate
LMICs: Low- and middle-income countries
REDCap: Research Electronic Data Capture
RR: Respiratory rate
SMPV: Sampov Meas Provincial Hospital
WHO: World Health Organization
