# Supplementary material for: Characteristics and outcomes of pediatric patients presenting at Cambodian referral hospitals without appointments: an observational study
Source: Int J Emerg Med. 2018 Mar 13;11:17. doi: 10.1186/s12245-018-0172-0 (PMC5849596; doi:10.1186/s12245-018-0172-0)
Supplement: Supplementary file 1 — Figure S1. Chief complaints. All chief complaints reported by patients were coded by researchers as one of the complaints listed. (PDF 1287 kb) [file 12245_2018_172_MOESM1_ESM.pdf]

S1 Fig

Chief Complaints

Top 15 Chief Complaints	Code
Abdominal pain	101
Chest pain	102
Diarrhea	103
Dizziness/vertigo	104
Fall	105
Fever	106
Genito-urinary problem	107
Headache	108
Laceration	109
Orthopedic injury	110
Respiratory problems	111
Traumatic injury	112
Skin complaint/trauma	113
Vaginal bleeding	114
Vomiting	115

A-E	Code
Abdominal problems	116
Abnormal behavior	117
Abuse	118
Alcohol related	119
Allergic reaction/ Anaphylaxis	120
Altered mental status	121
Assult	122
Back pain	123
Bites	124
Blast injury/explosion	125
Body aches	126
Burns	127
Cardiac arrest	128
Cardiovascular complaint	129
Crying/irritability	130
Convulsions/seizures	131
Dental/toothache	132
Diabetic problems	133
Drowning	134
Ear/nose/throat problem	135
Eye problem	136
Flank pain	137
Fluid/nutrition	138

F-Z	Code
Foreign body	139
Follow up	140
Gastrointestinal bleeding	141
Gunshot wound	142
Gynecological problem	143
Hemorrhage	144
Hypertension	145
Hyperthermia-Environmental	146
Hypothermia-Environmental	147
Industrial/machinery accident	148
Inhalation (smoke)	149
Infection	150
Joint pain	151
Land mine injury	152
Medication refill	153
Neck pain	154
Needlestick	155
Neurological complaint	156
Obstretrical problem	157
Other	158
Overdose (intentional)	159
Peripheral vascular pain	160
Pelvic pain	161
Procedure	162
Psychiatric/social problems	163
Rape	164
Rash	165
Scrotal Pain/Male GU	166
Shock	167
Stabbing	168
Sting	169
Stroke/CVA	170
Substance abuse	171
Syncope/fainting	172
Traffic injury-inside vehicle	173
Traffic injury-pedestrian	174
Unconsciousness	175
Unknown problem/Man down	176
Weakness	177
