# Supplementary material for: Characteristics and outcomes of pediatric patients presenting at Cambodian referral hospitals without appointments: an observational study
Source: Int J Emerg Med. 2018 Mar 13;11:17. doi: 10.1186/s12245-018-0172-0 (PMC5849596; doi:10.1186/s12245-018-0172-0)
Supplement: Supplementary file 2 — Table S1. Criteria for abnormal vital signs. (PDF 171 kb) [file 12245_2018_172_MOESM2_ESM.pdf]

Table S1 Criteria for abnormal vital signs

Age		Pulse		SBP		RR	
**lower limit**	**upper limit**	**lower limit**	**upper limit**	**lower limit**	**upper limit**	**lower limit**	**upper limit**
0 mos	<3 mos	<=100	>=150	<=65	>=85	<=35	>=55
3 mos	<6 mos	<=90	>=120	<=70	>=90	<=30	>=45
6 mos	<12 mos	<=80	>=120	<=80	>=100	<=25	>=40
12 mos	< 2 yrs	<=70	>=110	<=80	>=105	<=20	>=30
2 yrs	<5 yrs	<=60	>=140	<=80	>=110	<=20	>=34
5 yrs	<10 ys	<=60	>=140	<=80	>=110	<=18	>=30
10 yrs	<13 yrs	<=50	>=100	<=90	>=110	<=18	>=30
13 yrs	up	<=50	>=100	<=90	>=140	<=10	>=28

SBP = systolic blood pressure; RR = respiratory rate

Criteria for abnormal vital signs were selected by the authors based on multiple references, including:

Kliegman RM, Stanton BF, St. Geme J, Schor NF, Behrman RE.*Nelson textbook of pediatrics*, 20th ed. Philadelphia, PA: Elsevier; 2016.

Mahadevan SV, Garmel GM. An introduction to clinical emergency medicine. New York: Cambridge University Press; 2005.

Los Angeles County Department of Public Health. *PEDIATRIC SURGE POCKET GUIDE. http://publichealth.lacounty.gov/eprp/docs/emergency%20plans/pediatric%20surge%20pocket%20guide.pdf (accessed 04 March 2016).*
